# The Pathophysiological Impact of HLA Class Ia and HLA-G Expression and Regulatory T Cells in Malignant Melanoma: A Review

**DOI:** 10.1155/2016/6829283

**Published:** 2016-11-23

**Authors:** Lasse Lindholm Johansen, Jørgen Lock-Andersen, Thomas Vauvert F. Hviid

**Affiliations:** ^1^Centre for Immune Regulation and Reproductive Immunology (CIRRI), Department of Clinical Biochemistry, Zealand University Hospital, Roskilde, Denmark; ^2^Department of Clinical Medicine, University of Copenhagen, Copenhagen, Denmark; ^3^Department of Plastic Surgery, Zealand University Hospital, Roskilde, Denmark

## Abstract

Malignant melanoma, a very common type of cancer, is a rapidly growing cancer of the skin with an increase in incidence among the Caucasian population. The disease is seen through all age groups and is very common in the younger age groups. Several studies have examined the risk factors and pathophysiological mechanisms of malignant melanoma, which have enlightened our understanding of the development of the disease, but we have still to fully understand the complex immunological interactions. The examination of the interaction between the human leucocyte antigen (HLA) system and prognostic outcome has shown interesting results, and a correlation between the down- or upregulation of these antigens and prognosis has been seen through many different types of cancer. In malignant melanoma, HLA class Ia has been seen to influence the effects of pharmaceutical drug treatment as well as the overall prognosis, and the HLA class Ib and regulatory T cells have been correlated with tumor progression. Although there is still no standardized immunological treatment worldwide, the interaction between the human leucocyte antigen (HLA) system and tumor progression seems to be a promising focus in the way of optimizing the treatment of malignant melanoma.

## 1. Introduction

Cutaneous malignant melanoma is a type of cancer that develops in the melanocytes of the skin. The epidermis, which is the barrier of the body, that protects us from the outer environment, is made up of different types of cells, primarily squamous cells, basal cells, and melanocytes. In addition, the skin also contains important immune cells. Melanin is produced in the melanocytes and is the pigment that gives the skin its characteristic color, and it is in these cells that the malignant melanoma originates from; the tumors are frequently strongly pigmented. Another type of skin cancer is nonmelanoma skin cancer, which includes basal cell carcinoma and squamous cell carcinoma. These types of cancers are very common; however, they metastasize rarely. Unfortunately, there has been an annual increase in the incidence of malignant melanoma among different populations ranging from 3% to 7%, which corresponds to a doubling of rates every 10–20 years [1]. Worldwide, the highest incidence rates have been reported in Australia and New Zealand with incidence rates as high as 60 cases per 100,000 inhabitants per year [2]. Throughout Europe, age-standardized incidence rates in 2012 have been estimated to 11.4 per 100,000 for males and 11.0 per 100,000 for females. These have ranged from six new cases per 100,000 in Central and Eastern Europe, 10 cases in Southern Europe, and 19 cases in Northern Europe [3].

The median age is 62 years in the US, when the disease is detected for the first time. However, the disease also affects younger people under 30 and is one of the most common cancers among young people [4]. However, some studies from Australia, New Zealand, USA, several Western European, and Nordic countries have indicated a stabilization in the incidence rates in both sexes mainly among young people, and an increase in the incidence of malignant melanoma in the age group > 60 years [5, 6]. Based on data from 39,000 patients, the Amercian Joint Committee on Cancer calculated the five- and ten-year survival rates according to the TNM classification system: these were for clinical stage I (histological tumor, thickness ≤ 1 mm and node negative) 97% and 93% (resp., five- and ten-year survival rates), stage II (tumor thickness >1 mm and node negative) 53% and 39%, and stage III (with regional lymph node metastases) 46% and 33%. Additional important tumor factors are ulceration and mitotic rate [7].

## 2. Clinical Evaluation and Risk Factors

Regarding anatomic localization, the back has traditionally been the predilection site in males and the legs in females, with a tendency in recent years to a change in female presentation towards a male pattern [8, 9]. Malignant melanoma can arise from normal skin, benign nevi, and dysplastic nevi, where dysplastic nevi can be considered as an intermediate stage.

Of other risk factors, in addition to dysplastic nevi, UV radiation plays a very important role in the incidence of malignant melanoma creating cellular lesions in the DNA, pyrimidine dimers of C-T mutations [10]. Recent research has shown that C-T mutations are frequently found in malignant melanomas. However, these mutations are also seen in pancreatic cancer; therefore, it is uncertain whether they are directly connected to UV-radiation [11, 12].

Melanoma is related to intermittent sun exposure as well as to accumulated sun exposure. Twice the risk of developing malignant melanoma has been seen in individuals with skin type I or II compared to skin type III or IV. Skin type I is defined as always sunburned, never tanned, and skin type IV as never sunburned, always tanned [13–15]. However, the relationship between the UV radiation and malignant melanoma is very complex, as it is pointed out that chronic exposure to UV radiation in subjects with outdoor work has a protective effect against the development of malignant melanoma [13–17]. This complex relationship is exposed further, as malignant melanomas develop not only on the more UV-exposed areas of the body, but also on the trunk, most often in men, and on the lower extremities, most often in women. This may suggest that it is not an accumulated amount of UV radiation that is important for the malignant development and, in addition, it may indicate that there is a significant genetic factor involved [8, 18]. Individuals with many nevi may have a greater lifetime risk for the development of malignant melanoma.

## 3. Diagnosis, Treatment, and Prognostic Evaluation

The principles of treatment of localized malignant melanoma consist primarily of surgical intervention. However, medications are also in use when treating advanced disease. The efficacy of interferon-*α* has been investigated in several studies, and it was shown that high doses of interferon-*α* could delay the time of the first distal metastasis; however, that delay did not have a positive effect on overall survival [19]. In a more recent prospective study, the role of high-dose interferon-*α*2b therapy, or completion lymph node dissection, for patients with melanoma, staged by sentinel lymph node biopsy, was evaluated in patients, enrolled between 1997 and 2003, with 71 months' follow-up. No positive effect on disease-free survival or overall survival was identified for adjuvant therapy with high-dose interferon-*α* in patients with a single tumor-positive sentinel lymph node [20]. Other studies have found adjuvant treatment with interferon to have an overall positive effect on survival. Garbe et al. found that low dose (3 MU) interferon-*α*2a, administrated subcutaneously, given three times a week for two years improved overall survival and disease-free survival in melanoma patients with first manifestation of metastasis in regional lymph nodes when compared to treatment with interferon combined with dacarbazine or observation alone [21]. Another study has found that one-year maintenance treatment with intermediate-dose adjuvant interferon-*α*2b improved relapse-free survival, but this was not the case with two-year maintenance therapy [22]. Due to the high toxicity of interferon-*α* this drug is not considered standard adjuvant therapy in some countries [23]. In a retrospective study by Hughes et al., treatment with high-dose interleukin-2 of 305 patients with either malignant melanoma or metastatic renal cell carcinoma was investigated. Twenty-five per cent of the patients with metastatic melanoma or renal cell carcinoma achieved stable disease, defined as less than 20% progression in the disease, and not higher than 30% progression in the disease, after initial treatment with interleukin-2. This was associated with improved overall survival compared with patients, who had progressive disease. A disease control rate (DCR) was listed as the percentage of patients, whose disease did not progress after initial treatment. If the stable disease was taken into account, the treatment with interleukin had a DCR of 37.7%, and the study pointed out that this rate was more indicative than previously reported response rates of 15–20%, which underestimated the effect of the treatment [24]. Several medications, such as dacarbazine, vemurafenib, dabrafenib, and trametinib, are being used in the treatment of metastatic melanoma, but it seems that there is still a need for drugs with better long-term effects and less toxicity [25]. Ipilimumab, a recombinant, monoclonal antibody that interacts with and blocks the cytotoxic T lymphocyte associated protein 4 (CTLA-4) receptor in activated T cells, was the first systemic treatment that showed an improvement in survival in a phase III trial, treating patients with advanced melanoma. In 2010, a phase III trial consisting of 676 patients with stage III-/IV advanced melanoma showed a significantly better median survival when the treatment with ipilimumab (10.1 months) was compared with the tumor-associated antigen (TAA) glycoprotein (gp100) vaccine (6.4 months) [26]. Ipilimumab and dabarbazine, combined, have also been shown to have a positive effect on median survival when compared to dacarbazine combined with placebo [27]. The TAA gp100 was one of the first identified. The gp100 epitope peptide is restricted to HLA-A^*∗*^2402 and has been tested in clinical trials to treat melanoma patients [28]. The positive results from the treatment with ipilimumab lead to the approval in the US by the Food and Drug Administration in 2011 and later by the European Medicines Agency [26, 27]. Of other drugs that have shown promising results in the treatment of melanoma are the programmed cell death protein 1 (PD-1) inhibitors, nivolumab, and pembrolizumab. The PD-1 is a receptor located on T cells that, by binding to the PD-L1 and PD-L2 ligands, prevents the activation of the T cell. In different types of cancer, these ligands are upregulated and expressed in the tumor microenvironment, which suppress the activation of the T cells [29]. The recombinant monoclonal antibody, nivolumab, has in a recent study shown significant improvements in overall survival and progression-free survival, as compared with dacarbazine, among previously untreated patients with metastatic melanoma without a B-Raf protooncogene, serine/threonine kinase (BRAF) mutation [30]. Interestingly, in a study of melanoma cell lines and patient samples, Johnson et al. found evidence that melanoma-specific HLA class II expression may function as a marker for predicting response to anti-PD-1/PD-L1 therapy [31].

## 4. The Effect of UV Radiation

UV radiation, which damages the DNA, results in an increase of the production of melanin and blocks the cell cycle through microphthalmia associated transcription factor (MITF). This blockage of the cell cycle occurs to prevent unrestrained progression of melanocytes until the DNA is no longer being damaged [32]. Melanocortin-1 receptor (MC1R), a previous link to the activation of MITF, is activated by melanocyte-stimulating hormone (MSH) which is activated by UV radiation.

MC1R, a gene with high genetic polymorphism, has a large influence on the pigmentation of the skin in every individual. There are a number of recessive MC1R alleles, which have a high penetrance in individuals with red hair. These individuals have a reduced ability to increase the UV-induced pigmentation [33]. Moreover, these alleles are associated with malignant melanoma with an odds ratio (OR) of 1.4–2.4 [34]. Germline mutations in cyclin-dependent kinase inhibitor 2A (CDKN2A), a kinase that plays a role in UV-induced melanin production by encoding two different tumor suppressor genes, p16INK4a and p14ARF, are associated with familial melanoma [35]. These mutations have been observed in 10% of families with two cases of malignant melanoma and 30–40% of families with three or more cases of malignant melanoma [36]. High-penetrance mutations in a kinase, cyclin-dependent kinase 4 (CDK4), which also plays a role in the melanogenesis by inducing the progression of the cell cycle, are also observed [37]. BRAF, a member of the raf family, is a protein kinase that is encoded by the BRAF gene. This protein kinase is a part of mitogen-activated protein kinase pathway (MAPK signaling pathway) of melanocyte proliferation, and through the MAPK signaling pathway, BRAF regulates a variety of cellular processes, such as growth, proliferation, and apoptosis. Mutations in BRAF result in an alteration of the activity of the protein kinase, which hereby acts as an oncogene. This oncogene can give rise to benign and malignant neoplasms. The vast majority of mutations in BRAF are V600E mutations, in which glutamic acid (E) is replaced with valine (V) at codon 600 and causes the protein to become overactive [38]. The benign and malignant changes this transformation may give rise to are due to an increase in cell proliferation and survival [32, 39, 40]. As previously mentioned, malignant melanomas are more commonly found in individuals with skin type I-II, compared to skin type III-IV. A higher incidence of malignant melanomas is also seen in red-haired compared to dark-haired individuals [41]. Overall, in epidemiological studies, a higher risk of malignant melanoma is observed by a reduction in pigmentation and an increased number of nevi [32, 41, 42].

## 5. Immunological Mechanisms in Malignant Melanoma

**Figure 1:**
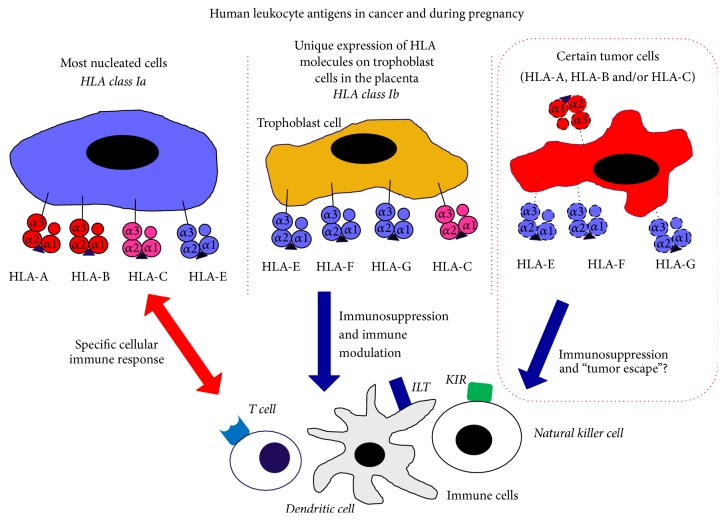
In some cases, tumor cells, for example, malignant melanoma cells, may obtain through selection processes an HLA expression profile that in varying degrees mimics the HLA expression on extra-villous trophoblast cells at the fetomaternal interface during pregnancy. The specific HLA expression profile on tumor cells involving one or several of the HLA class Ib molecules may be one mechanism leading to immunosuppression and immune escape (ILT: immunoglobulin-like transcript; KIR: killer-cell immunoglobulin-like receptors).

Since the 1950s, the physiological function of the adaptive immune system in relation to cancer, understood as the individual's own defense against the growth of transformed cells, has been increasingly explored. This function is known as* immune surveillance*. Growing tumors may present different molecules that are recognized as foreign antigens and are therefore defeated by the immune system. These tumor antigens are recognized by CD8^+^ T cells that differentiate into cytotoxic lymphocytes (CTL) and fight the transformed cells. Tumor antigens are presented by the major histocompatibility complex (MHC) class I molecules on dendritic cells, known as antigen-presenting cells (APC). The major histocompatibility complex in humans is named the human leukocyte antigen (HLA) system (Figure 1). In order for the differentiation of naive CD8^+^ T cells to the CD8^+^ CTL to take place, there must also be a costimulation and/or help from CD4^+^ T cells that bind to MHC class II molecules. By APC B7 costimulation secondary signals help the activation of the CD8^+^ CTL. The binding of CD4^+^ T cells to the MHC releases cytokines that contribute to the differentiation process.

**Figure 2:**
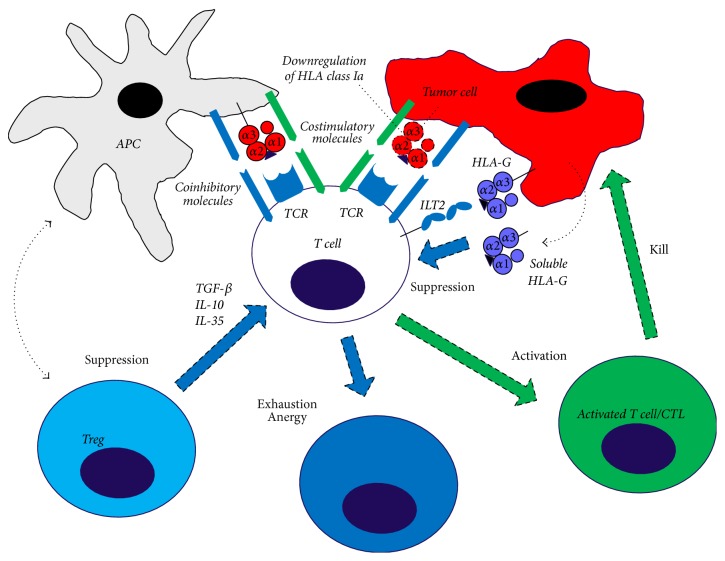
Schematic representation of immunological mechanisms discussed in the text that may lead to escape of malignant melanoma cells from immune surveillance. The expression of HLA class Ia molecules on the tumor cell surface may be compromised or downregulated. The tumor cell may begin, or may be selected, to express immunosuppressive HLA-G molecules that exist in both membrane-bound forms and soluble forms. Regulatory T cells in the tumor microenvironment secrete TGF-*β*, IL-10, and IL-35 that inhibit T cell functions. If the immune checkpoint balance is in favor of negative signals, it will result in inhibition of T cell responses with exhausted T cells and cytotoxic T lymphocytes in anergy (APC: antigen-presenting cell; CTL: cytotoxic T lymphocyte; IL-10: interleukin-10; IL-35: interleukin-35; ILT2: immunoglobulin-like transcript-2; TCR: T cell receptor; TGF-*β*: transforming growth factor-*β*; Treg: regulatory T cell).

However, it is not always the case that the foreign tumor cells are suppressed as they may prevent the presentation to the CD8^+^ T cells. This phenomenon is known as* immune escape*, which may take place in different ways: lack of expression of the tumor antigen, lack of expression of the MHC class I molecules, production of inhibitory cell surface proteins, or production and secretion of cytokines (Figure 2). Since the tumor cells have been able to develop these escape mechanisms, the hypothesis of* immune editing* was established, which consists of three parts: elimination, steady state, and escape [43]. In the first step, the tumor cells are recognized and killed by the immune response, as described earlier. In case of tumor variants that are not eliminated by the immune response, the immune system retains the growth of these cells throughout the life of the host and thus achieves steady state. At the final stage, escape, tumor cells avoid elimination and achieve tumor escape [44]. In cases where the tumor cells do not express MHC class I, the natural killer (NK) cells play an important role. All healthy human, eukaryotic cells express MHC class I at the cell surface as well as ligands for activating NK cells. NK cell inhibitory receptors react with the expressed MHC class I molecule, thus avoiding the lysing of the healthy cells. As mentioned, certain tumor cells downregulate their expression of MHC class I, which protects them from degradation by CD8^+^ CTL. In the cases where MHC class I is downregulated, the NK cells are activated by the activating ligands on the surface of the tumor cell because they do not express MHC class I, which would otherwise react with the NK cell inhibitory receptors. In this way, the tumor cell is lysed even though it has achieved immune escape from the CD8^+^ CTL. In the 1950s, it was shown in mice studies that the rejection of grafted tissue was coupled to the adaptive immune system. This was due to foreign antigens on the surface of the transplanted cells and foreign variants of surface proteins, especially MHC molecules, which mainly the T cells responded to, and a rejection of the transplanted tissue was mediated. MHC/HLA proteins are extremely polymorphic; there are a very high number of alleles, more than 1,000 for some of the genes. This makes it very unlikely that two randomly selected individuals may be able to function as donor and recipient, because the chance that they have two identical sets of MHC proteins is very small.

The tumor microenvironment has for many years been a point of interest in melanoma research. The melanoma cells interact with the microenvironment in many different ways, for example, through cell-matrix contact and through secreted growth factors and cytokines. In order for the melanoma cells to successfully migrate and invade, the cells need to activate growth factors that regulate cell-adhesion [45]. Basic fibroblast growth factor (bFGF), a growth factor that is produced and secreted in melanoma cells, which promotes proliferation and survival in human melanocytes in an autocrine manner, has been described to correlate with melanoma tumor progression. Furthermore, overexpression of bFGF enhances the proliferation of melanocytes and anchorage-independent growth [46]. The melanocytes that overexpress bFGF can, without the presence of insulin-like growth factor (IGF-1) and melanocyte-stimulating hormone (MSH), grow and proliferate, and the bFGF secreted by melanoma cells can stimulate proliferation of stromal cells in a paracrine manner [47]. Besides bFGF, other melanoma-secreted growth factors, such as platelet-derived growth factor (PDGF) and transforming growth factor- (TGF-) *β*, seem to induce proliferation and activation of fibroblasts and endothelial cells and in this way exercise paracrine functions in angiogenesis and stroma formation. The effects of PDGF stimulate the production of collagen and the glycoproteins, fibronectin and laminin, by neighboring fibroblasts [48]. PDGF, secreted by melanoma cells, furthermore stimulates the production and secretion of IGF-1, which stimulates the proliferation of melanoma cells. The promotion of melanoma growth is enhanced by the release of bFGF and endothelin by activated fibroblasts [49]. A well-known mechanism in tumor progression is angiogenesis. In melanoma cells, this process is stimulated by both autocrine and paracrine growth factors, such as vascular endothelial growth factor (VEGF), bFGF, PDGF, and TGF-*β*, and the progression of the melanoma cells leads to increased levels of VEGF and bFGF [45, 50]. A member of the VEGF family produced by melanoma cells, placental growth factor (PIGF), binds to neuropilin-1 and neuropilin-2 receptors on endothelial cells, which, in synergy with VEGF, exert the angiogenic actions of the endothelial cells. The prognosis and tumor progression have been correlated with levels of PIGF, and a study by Fischer et al. has suggested that treatment against PIGF could be a potential target when developing novel anticancer therapies [51]. The secretion of interleukin-8 (IL-8) by endothelial cells has an effect on vascular permeability and stimulates the migration of melanoma and endothelial cells [50]. UV radiation (UVB), TGF-*β*1, and hypoxia can induce the expression of IL-8 in melanoma cells, potentially increasing their metastatic potential, and a study by Ugurel et al. suggested that increased levels of serum IL-8 in melanoma patients were correlated with advanced disease and poor overall survival [52]. Understanding the tumor microenvironment is essential in the ongoing development of treatments for melanoma, as well as other types of cancer, as it makes the foundation of knowledge required to target the many different molecular mechanisms in tumor progression.

## 6. HLA Class Ia Molecules in Malignant Melanoma

Major histocompatibility complex molecules are divided into two classes, classes I (which is subdivided into Ia and Ib) and II. The MHC class Ia, known as the classical class I molecules, is composed of HLA-A, HLA-B, and HLA-C and class Ib of HLA-E, HLA-F, and HLA-G [53].

HLA class Ia molecules consist of three subunits, a HLA class I heavy chain, *β*_2_-microglobulin (*β*_2_m), and a peptide [54]. In order for these molecules to be expressed on the cell surface, they have to undergo assembly in the endoplasmatic reticulum followed by a transport to the cell surface. This process requires the transporter associated with antigen processing 1 (TAP1), TAP2, and different ER-resident chaperons such as calnexin, calreticulin, and tapasin [55, 56]. The heavy chain-*β*_2_m heterodimer is stabilized through noncovalent protein-protein interactions by *β*_2_m, which makes the binding of endogenous antigenic peptides possible with the help from TAP, and thereby allows for the assembled heavy chain-*β*_2_m-peptide trimeric complexes to be transported to the cell surface, where they are recognized by CTL [57]. The expression of HLA class Ia molecules on the tumor cells may be modulated by cytokines in the tumor microenvironment. One example would be interferon-*α* (IFN-*α*) and interferon-*β* (IFN-*β*) that upregulate HLA class Ia expression.

It appears that low or no expression of HLA class Ia results in a poorer prognosis for patients with cervical cancer, rectal cancer, and melanoma patients with advanced disease [58–60]. However, it has also been shown that a total loss of HLA class Ia expression is correlated with improved survival in colorectal cancer patients [61]. The downregulation of HLA class Ia molecules has been shown to be correlated with a poorer response to immunotherapy of malignant melanoma patients in one study [62]. Tumors are known to downregulate the expression of HLA class Ia molecules, resulting in a significant tumor escape mechanism. This is due to a change in the interaction between tumor cells and specific T cells and NK cells [63–66]. In a study by Carretero et al., the amount of expressed HLA class Ia protein for ten metastatic lesions from a melanoma patient, who was under immunotherapy treatment, was measured. Two out of ten lesions showed tumor progression and had a low expression of HLA class Ia. The eight other lesions showed tumor regression and expressed a high amount of HLA class Ia [58]. In patients with rectal cancer, there has been demonstrated a higher amount of stage IV tumors in patients with a loss of HLA class Ia expression [60]. It has been suggested that immunotherapy leads to an alteration of the tumor microenvironment promoting a release of immune-stimulating factors by immune cells, which leads to an upregulation of HLA class Ia expression in tumor cells, eventually leading to the recognition and destruction of the tumor cells by antigen-specific T cells. However, if the tumor cells bear irreversible defects in the HLA class Ia genes, the antigen presentation remains defective after immunotherapy leading to tumor immune escape due to an impairment of the amplification of the immune response [67, 68].

Large series of tumor lesions from solid tumors of melanoma, colorectal, bladder, head and neck, breast, kidney, lung, prostate, and cervical carcinoma have shown a defect in HLA class Ia expression [69–72]. Depending on the tumor type, the downregulation, loss of HLA loci, and HLA class I allospecificities have ranged between 3.4% and 60%. The loss of heterozygosity at the HLA loci is frequent in tumors such as melanoma and colorectal carcinomas, but not in renal cell carcinoma, and this loss of heterozygosity could therefore contribute to the downregulation of HLA class Ia in specific types of tumors [73]. Total loss of HLA class Ia by tumor cells has been found to alter the immunological response against tumor cells due to resistance to recognition by CTL [74]. The formation of HLA class I heavy chain-*β*_2_m-peptide complexes and the transport to the cell surface requires *β*_2_m, and a loss of *β*_2_m is frequently found in phenotypes, where loss of HLA class Ia is observed [75, 76]. The underlying mechanism for this loss of *β*_2_m in malignant cells has been found to be due to loss of heterozygosity, mutations in one copy of the *β*_2_m gene, and loss of the other copy [76]. In a study by Chang et al., where the molecular defects underlying HLA class I loss in five melanoma cells lines derived from recurrent metastases following initial clinical response to T cell-based immunotherapy were characterized, has suggested the emergence of mutations in the *β*_2_m gene following strong T cell-mediated immune selection [77]. An interesting point was that the development of multiple escape mechanisms by melanoma cells to avoid T cell-mediated selective events might be reflected by multiple HLA class I defects within the tumor cell population [77].

## 7. HLA-G and Regulatory T Cells in Cancer and Pregnancy

Human leukocyte antigen-G, which is one of the nonclassical MHC class Ib molecules, can be detected in fetal tissue such as the amniotic sac, cell precursors, and cytotrophoblasts. HLA-G has basically a similar structure to HLA class Ia molecules but is, in contrast to these, known for a low genetic diversity [66]. In adults, HLA-G is found in the cornea, thymus, pancreas, endothelial cell precursors, and erythroblasts [78]. In addition, HLA-G is also expressed in antigen-presenting cells (APC) and macrophages. Overall, HLA-G is found in two different forms: membrane-bound HLA-G (G1–G4) and secreted isoforms (G5–G7) [79]. Human leukocyte antigen-G has an important tolerance-inducing function and can modulate the immune system by binding to inhibitory receptors on lymphoid cells, NK cells, dendritic cells, macrophages, and monocytes. HLA-G binds to three receptors: immunoglobulin-like transcript 2 (ILT2), immunoglobulin-like transcript 4 (ILT4), and killer immunoglobulin-like receptors 2DL4 (KIR2DL4). The ILTs are inhibitory, and KIR2DL4 possesses activating properties as well [80–83]. By binding to these inhibitory receptors, HLA-G may induce tolerance in various ways, such as differentiation, proliferation, cytokine secretion, and cytolysis of the normal immune response [79]. The complex between HLA-G and APCs has a certain inhibitory effect on CD4^+^ T cells by inducing their differentiation into regulatory T cells [84]. Regulatory T cells are important to sustain immune tolerance and prevent autoimmune diseases. Using a process called trogocytose, wherein the plasma membrane and anchored proteins are transferred via cell-cell contact, the NK cells, dendritic cells, and T cells that receive membrane-bound HLA-G molecules from cancer cells, downregulate the immune response [85]. Thereby, HLA-G might contribute to a reduced immune response against the tumors of cancer patients [79]. Aberrant HLA-G expression has often been found in tumor lesions but is rare in adjacent “nontumor” tissue [73], and the expression of the nonclassical class Ib antigen in tumors has often been associated with tumor progression and a poor prognosis for cancer patients [86–89]. Studies have shown that HLA-G is expressed in a variety of cancers, such as hepatocellular carcinoma, gastric cancer, and breast cancer, and is correlated with a poor survival [90–92]. HLA-G expression has, besides in cancer, also been found in other pathological situations such as transplantation and viral infections [89, 93–95]. Both soluble and membrane-bound HLA-G have the ability to upregulate inhibitory receptors [96].

A high frequency of HLA-G surface expression and high serum HLA-G concentration has been measured in both hematological and solid tumors, and it has been shown that the high expression of HLA-G and sHLA-G is correlated with a poorer prognosis in cancer patients. Therefore, it might indicate that HLA-G plays an important role in the development of tumors by inducing immune escape [97]. Soluble HLA-G is secreted by both tumor cells and cells of the immune system, such as monocytes, T cells, and dendritic cells, and it is conceivable that sHLA-G levels can be used as a diagnostic tool to distinguish benign from malignant tumors [79, 98]. The most abundant expression of HLA-G in normal conditions is on the surface of trophoblast cells. It is important to understand how the trophoblast cells and HLA-G-expressing cancer cells are partly similar to each other (Figure 1). The manner in which cancer cells avoid the immune system of the host by immune escape may be comparable to the fetomaternal tolerance observed between the mother and the fetus consisting of semiallogenic cells. Human leukocyte antigen-G molecules expressed on trophoblast cells can effectively suppress the local immune response in the uterus so that the fetus is not recognized as a foreign organism to be combated [53, 66]. This would provide a better understanding of how the tumor cells may avoid the host immune system. It may partly be by some of the same mechanisms as the semiallogenic fetus is accepted by the pregnant woman.

The regulatory T cells have an important task of promoting and maintaining immune tolerance by inhibiting other effectors, such as helper T cells and cytotoxic T cells, and prevent an excessive T cell response in chronic infections. It has been shown that CD4^+^ and CD8^+^ T cells that were stimulated in the presence of HLA-G lost their ability to respond to antigenic stimulation and developed into regulatory T cells with the ability to inhibit other T cells [84]. A study by Baumgartner et al. evaluated the amount of regulatory T cells in advanced melanoma disease and found that higher levels of regulatory T cells were correlated with a worse outcome in patients with advanced malignant melanoma, and this might probably be due to a negative effect on the antitumor response [99, 100]. Therefore, it can be speculated that high expression of HLA-G, as well as regulatory T cells, might contribute to a poor prognosis in patients with malignant melanoma. It has been difficult to characterize and study the regulatory T cells due to lack of biomarkers. However, it has been shown that these cells express the transcription factor Foxp3, which can both be used as a biomarker and also as an overall target for their development.

## 8. HLA-G in Malignant Melanoma: A Role for Cancer Immune Therapy Based on HLA-G?

A number of studies have investigated HLA-G protein expression in malignant melanoma. Controversies exist regarding the detection of HLA-G protein expression in melanoma tumor biopsies, while nearly all melanoma cell lines seem to be negative [101–105]. These controversies might be due to the use of different monoclonal antibodies, different technical procedures, and low number of samples in some studies. However, in general, approximately 30% of surgically removed lesions or biopsies are positive for HLA-G protein [102, 105, 106]. HLA-G expression seems to be correlated with malignant transformation and a worse prognosis with relapse or metastasis in some studies [104–106]. However, these are all small studies. Furthermore, soluble HLA-G serum levels have been reported to be elevated in melanoma patients [107].

Based on a mouse model it has been shown that HLA-G-positive tumor cells develop and tolerize the host antitumor immune response* in vivo* [108]. The xenotumor model involves the injection of human tumor cells (M8) transfected with HLA-G subcutaneously in immunocompetent mice. The model works because HLA-G can bind and mediate signals via the murine receptor paired immunoglobulin-like receptor-B (PIR-B), which is the homolog of human ILTs [108]. With the use of this model it was demonstrated that human tumor cells expressing HLA-G can grow in an immunocompetent host and that it affects both the innate and the adaptive immune system. The main mechanisms for the tumor escape mediated by HLA-G were an expansion of blood myeloid-derived suppressor cells (MDSCs), loss of peripheral CD4^+^ and CD8^+^ T cells, and a cytokine profile in favor of Th2 versus Th1/Th17 [108]. Interestingly, it was possible to inhibit the development of the tumor* in vivo* with the administration of a specific anti-HLA-G blocking antibody. This opens for the possibility for considering HLA-G as an immune checkpoint molecule, and blocking the function of HLA-G may be a new therapeutic strategy in cancer immunotherapy.

Results from an* in vitro* study of samples from renal cell cancer patients indicate that HLA-G-peptide-based cancer immunotherapy may be possible [109]. Several peptides derived from the HLA-G molecule were tested based on the binding motif to HLA-A24 alleles. One peptide, HLA-G_146–154_, was observed to effectively induce peptide-specific CTLs and these exhibited cytotoxic activity against HLA-G-expressing HLA-A24-positive renal cell carcinoma cells [109]. Furthermore, a recent study showed that a MHC class II-binding peptide, HLA-G_26–40_, was effective in eliciting a tumor-reactive CD4^+^ T cell response [110]. It will be interesting in the future to explore the opportunities for modulating HLA-G expression in melanoma tumor cells and other tumors or induce an HLA-G peptide-specific immune response as new innovative cancer immunotherapy.

## 9. Conclusion

The incidence of malignant melanoma has been increasing through the years. Skin type, the number of nevi and dysplastic nevi, and sun exposure are among the currently well-known risk factors in the development of this type of cancer. Several medications are being used in the treatment of the disease but have not yet been able to substitute for surgical excision as a stand-alone treatment. The effects of UV radiation are well known, and several mutations, such as CDKN2A and BRAF mutations, have been shown to correlate with malignant melanoma. The focus on and the exploration of the immunological side of the pathophysiology have, with an advance in medical technology, increased through the years, and correlations between the prognosis and the human leucocyte antigen (HLA) system have been described. A loss or downregulation of HLA class Ia has been seen to have a negative impact on the prognosis of malignant melanoma but has been seen to have a positive impact on the prognosis of other types of cancer. A high expression of HLA-G and regulatory T cells have both, separately, been shown to worsen the outcome of malignant melanoma. More studies are needed for a better understanding of the complex mechanisms behind the impact of HLA classes Ia and Ib on the prognosis in order to further advance the current diagnostic tools and treatment of the disease.
